# Using Transcriptome Analysis to Identify Genes Involved in Switchgrass Flower Reversion

**DOI:** 10.3389/fpls.2018.01805

**Published:** 2018-12-04

**Authors:** Wang Yongfeng, Zheng Aiquan, Sun Fengli, Li Mao, Xu Kaijie, Zhang Chao, Liu Shudong, Xi Yajun

**Affiliations:** ^1^College of Agronomy, Northwest A&F University, Yangling, China; ^2^State Key Laboratory of Crop Stress Biology for Arid Areas, Yangling, China; ^3^Yangling Vocational & Technical College, Yangling, China; ^4^Institute of Cotton Research, Chinese Academy of Agricultural Sciences, Anyang, China

**Keywords:** switchgrass, floral reversion, cytokinin, flower maintenance, transcriptome

## Abstract

Floral reversion is a process in which differentiated floral organs revert back to vegetative organs. Although this phenomenon has been described for decades, the underlying molecular mechanisms remain unclear. In this study, we found that immature switchgrass (*Panicum virgatum*) inflorescences can revert to neonatal shoots when incubated on a basal medium with benzylaminopurine. We used anatomical and histological methods to verify that these shoots were formed from floret primordia through flower reversion. To further explore the gene regulation of floral reversion in switchgrass, the transcriptome of reversed, unreversed, and uncultured immature inflorescences were analyzed and 517 genes were identified as participating in flower reversion. Annotation using non-redundant databases revealed that these genes are involved in plant hormone biosynthesis and signal transduction, starch and sucrose metabolism, DNA replication and modification, and other processes crucial for switchgrass flower reversion. When four of the genes were overexpressed in *Arabidopsis thaliana*, vegetative growth was facilitated and reproductive growth was inhibited in transgenic plants. This study provides a basic understanding of genes regulating the floral transition in switchgrass and will promote the research of floral reversion and flower maintenance.

## Introduction

Flowering is an essential process in the angiosperm life cycle and facilitates the transmission of hereditary components to offspring via sexual reproduction (Scutt and Vandenbussche, 2014). Mature plants generally produce determinate blossoms and seeds (Irish, 2010). However, in some plant species, the differentiated floral meristems can also be reversed into a vegetative phase when the environment becomes adverse for flowering (Battey and Lyndon, 1990; Tooke et al., 2005; Wang et al., 2010; Sen et al., 2013). This ‘reverse development’ is known as flower reversion, and has been described in both dicots and monocots (Battey and Lyndon, 1990; Tooke et al., 2005; Trevaskis et al., 2007). Studying flower reversion has greatly contributed to our understanding of the molecular mechanisms involved in flower formation and flower maintenance (Tooke et al., 2005; Irish, 2010; O’Maoileidigh et al., 2014).

The first detailed description of floral reversion was published in 1990 (Battey and Lyndon, 1990). Since then, researchers have studied the underlying reversion mechanisms using mutants with distorted flower development (Scutt and Vandenbussche, 2014). In *Arabidopsis thaliana*, the *LEAFY* (*LFY*) gene is required for flower determination, and studies have further indicated it plays a significant role in suppressing flower reversion. Knocking out *LFY* and *Agamous* (*AG*) function in *A*. *thaliana* leads to floral meristem reversion under a short photoperiod (Okamuro et al., 1996). Studies have also indicated that *LFY* and *APETALA 1* (*AP1*) promote flower formation in *A*. *thaliana* by inhibiting the expression of *AGAMOUS-LIKE 24* (*AGL24*), a flower formation suppressor (Yu et al., 2004). The MADS-box genes are another group of important regulators in flower development (Fornara et al., 2003). Downregulated expression of the MADS-box gene *TM29* in tomato (*Solanum lycopersicum* L.) caused parthenocarpic fruit development and floral reversion (Ampomah-Dwamena et al., 2002). In maize (*Zea mays* L.), the *indeterminate 1* (*ID1*) gene was found to be a floral reversion suppressor, since the non-functional mutant *id1-m1* produced plantlets instead of florets on the tassel branches (Colasanti et al., 1998). These single mutant studies have contributed to our knowledge of flower reversion, yet a comprehensive understanding of gene expression patterns during flower reversion is still missing.

RNA sequencing (RNA-seq) is a powerful technique to examine transcriptome changes under specified spatial-temporal conditions and is especially useful for investigating insufficiently studied phenomena or species. In plants, RNA-seq has identified many important genes involved in metabolite biosynthesis (Wang et al., 2012; Yang et al., 2014), biotic and abiotic stress resistance (Liu et al., 2011), tillering (Palmer et al., 2012), flower development (Singh and Jain, 2014; Zhang et al., 2014), and fruit formation (Jiang et al., 2015).

Switchgrass (*Panicum virgatum*) is considered a model bioenergy crop due to its high biomass production and broad adaptability (Parrish et al., 2012). We found that switchgrass INFs underwent flower reversion when cultured on SIM (Chai et al., 2012), but produced flowers *in vitro* when cultured on Murashige and Skoog’s basal medium (MS; Murashige and Skoog, 1962). To identify the genes involved in switchgrass flower reversion, we compared the gene expression patterns of INFs incubated on SIM and MS media and uncultured INFs. Our primary aim was to gain an overall understanding of genes regulating flower reversion, which could benefit switchgrass breeding for high yields of biomass in the future.

## Results

### Evidence of Flower Reversion

**FIGURE 1:**
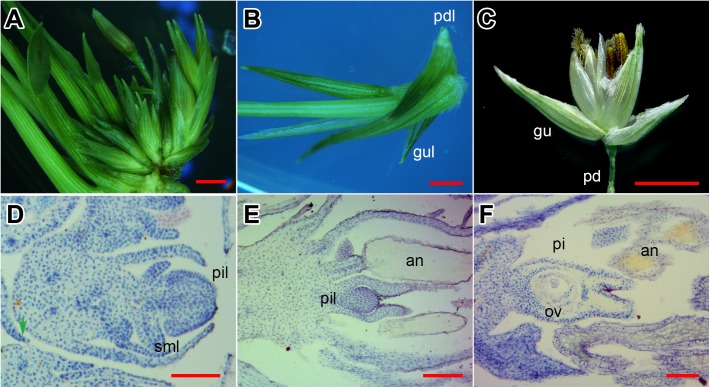
Evidence of flower reversion in switchgrass. **(A)** Reversed shoots and unreversed florets. **(B)** Single reversed shoot separated. **(C)** A normal floret from switchgrass plant. **(D)** Longitudinal section of a reversed shoot. **(E)** Longitudinal section of a half-reversed floret. **(F)** Longitudinal section of an unreversed floret. Pd, pedicel; pdl, pedicel-like; gu, glume; gul, glume-like; pi, pistil; pil, pistil-like; sml, stamen-like; an, anther; st, style; ov, ovule. **(A–C)** Bar = 2 mm; **(D,E)** bar = 0.5 mm.

The SIM culture induced neonatal shoots in switchgrass INFs (Figure 1A). As shown in Figure 1A, the emerged shoots were connected with unreversed florets, and seem to be derived from the same rachilla, yet the base was clearly separated. There were shortened pedicel-like organs at the bottom of shoots (Figures 1B,C). Moreover, two small leaves were bent inward at the base of the shoot (Figure 1B) and were similar to glumes of the floret (Figure 1C).

Next, we investigated the histological structure of the reversed florets (Figure 1D). In longitudinal sections of normal florets, the stamens and pistils were fully developed (Figure 1F) with separated styles and the ovule clearly visible (Figure 1F). In the half-reversed florets, the pistils were under-developed and spherical-shaped (Figure 1E); the stamens were normal (Figure 1E). In the reversed florets, the pistils developed into a deformed tissue without a style or stigma, and the stamens degenerated into pin-like tissues (Figure 1D). These results indicate that the emerged shoots on SIM were generated from floret primordia on the INF, and were most likely initiated from the pistil primordia.

### Flower Reversion of Switchgrass in Different Stages

**FIGURE 2:**
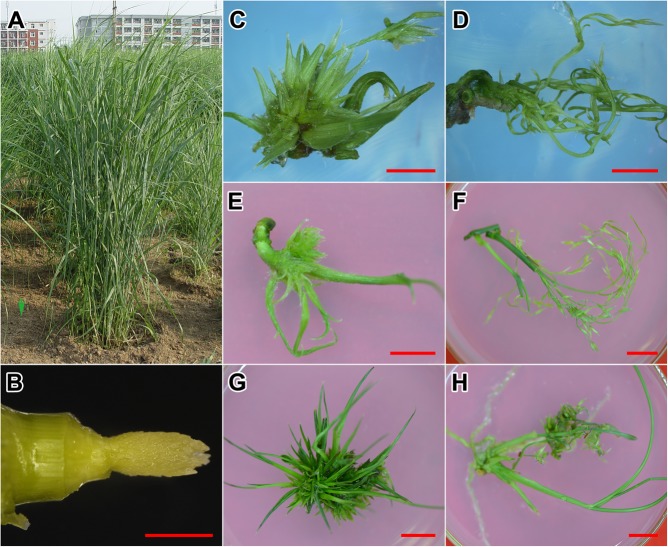
Incubation of the experimental material. **(A)** Switchgrass plants used in this research. **(B)** Immature inflorescences used for incubation. **(C)** Explant cultured on SIM medium for 20 days. **(D)** Explant cultured on MS medium for 20 days. **(E)** Explant cultured on SIM medium for 30 days. **(F)** Explant cultured on MS medium for 30 days. **(G)** Explant cultured on SIM medium for 60 days. **(H)** Explant cultured on MS medium for 60 days. Bars = 1 cm.

We harvested the 1 cm long INF at elongation stage 4 (E4; Figures 2A,B) (Moore et al., 1991; Burris et al., 2009). After 20 days of incubation on SIM medium, floret primordia were further developed, from which the compact, glume-like organs were formed (Figure 2C). In contrast, explants incubated on the MS medium developed stretched peduncles and young florets (Figure 2D). After 30 days, the glume-like organs on SIM further elongated and transformed into leaf-like tissues (Figure 2E). On MS medium, the peduncles elongated and the florets were initiated (Figure 2F). After 60 days of incubation, INFs were completely reversed into neonatal shoots on SIM medium (Figure 2G). Florets on MS medium developed into mature flowers with anthers and stigmas (Figure 2H).

### RNA Sequencing and Identification of Novel Transcripts

To investigate the molecular mechanism of flower reversion, we performed RNA-seq on reversing flowers on SIM medium (simplified as REV), *in vitro* flowers on MS medium (simplified as FLO), and uncultured immature inflorescences (simplified as INF). Considering that gene regulation of flower reversion occurred prior to the phenotypic alteration, we used explants incubated for 20 days in the subsequent transcriptome analysis.

Sequencing the switchgrass samples (*n* = 12) generated a total of 62.24 Gb of sequencing data, comprising 308.14 million clean reads. To guarantee accurate data analysis, we filtered out all low-quality reads and obtained 225.29 million high-quality reads from 45.51 Gb of data (Table 1). The high-quality reads were mapped to the Switchgrass reference genome^1^ (version 4.1) and assembled separately. The transcripts were merged to a uniform set of transcripts for all 12 samples. As a result, 63,915 loci containing 93,800 consensus transcripts were detected. Compared to the reference annotation, 33,499 loci were missed while 13,491 were newly detected (Supplementary Table S1). Statistical analysis showed that the percentage of missed loci ranged from 32.7 to 36.0%, and the percentage of novel loci ranged from 9.4 to 12.3% (Supplementary Table S1).

**Table 1:** Results of the raw data assessment.

Sample	ID	Raw data			Filtered data			Read alignments	
		Read_sum (million)	GC (%)	Q30 (%)	Read_sum (million)	GC (%)	Q30 (%)	Mapped_reads (million)	Map_rate (%)
REV	T1	25.0	55.6	80.0	17.7	52.6	91.5	16.6	94.0
	T2	26.3	54.1	80.2	19.4	51.9	91.6	18.1	93.0
	T3	25.8	55.7	80.2	18.7	52.5	91.5	17.6	94.2
	T4	26.1	55.8	80.2	20.1	53.3	91.3	19.0	94.7
FLO	T5	23.0	55.8	80.1	19.4	51.3	92.2	18.3	94.4
	T6	26.3	54.4	80.2	19.5	52.8	91.8	18.5	94.7
	T7	26.4	54.9	80.1	18.7	52.7	91.4	17.5	93.2
	T8	24.1	53.2	80.2	19.2	51.3	91.8	17.9	93.6
INF	T9	26,4	55.7	80.2	18.1	50.5	92.1	16.7	92.5
	T10	27.5	56.3	80.0	18.3	52.6	91.6	17.3	94.4
	T11	26.5	54.5	80.0	16.9	52.8	91.5	16.0	94.7
	T12	24. 6	55.6	80.1	19.4	51.5	91.9	18.2	93.7

*REV, material obtained by floral reverse induction on SIM medium; FLO, material obtain by in vitro flower induction on MS medium; INF, immature inflorescence without incubation. Four replications were included in each treatment.*

### Mining of Differentially Expressed Genes

**FIGURE 3:**
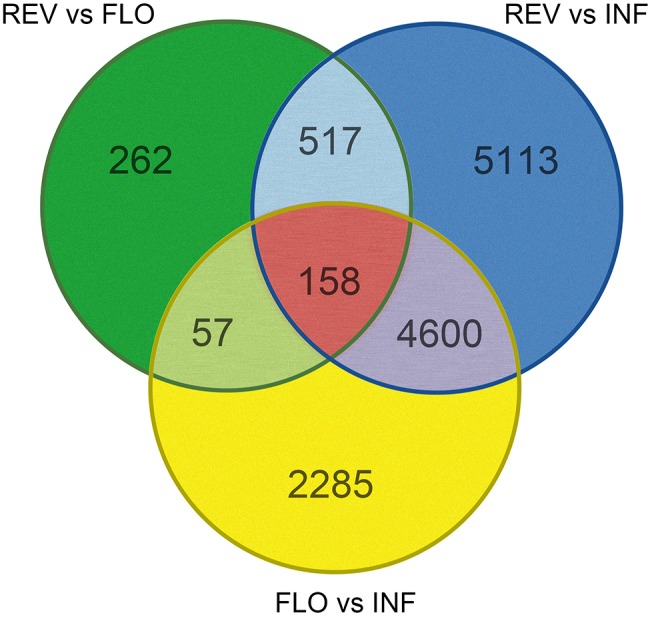
Distribution of differentially expressed genes (DEGs) in pairwise comparison.

Pairwise comparison between REV and FLO was expected to show differences in gene expression DEGs related to flower formation and reversion. Comparison of INF and REV was expected to generate DEGs related to floral reversion and the *in vitro* culture environment response, and comparison between INF and FLO was expected to generate DEGs related to *in vitro* flower development and *in vitro* culture environment response. To explore genes involved in switchgrass flower reversion, we combined results of the pairwise comparisons and used DEGs from REV vs. FLO and REV vs. INF, but not FLO vs. INF, as candidate genes (Figure 3).

Among all pairwise comparisons, REV vs. FLO produced the smallest number of DEGs (994), followed by FLO versus INF (7,100) and REV versus INF (10,388). The large difference in the number of DEGs of the three comparisons indicated that REV and FLO were the most similar materials, and *in vitro* culture resulted in a more dramatic change in expression patterns than flower reversion. Through the filtration approach described above, 517 genes were identified as significant in the induction of floral reversion. These genes are hereby referred to as ‘filtered-DEGs’ (Figure 3 and Supplementary Table S2).

Gene expression pattern analysis showed that 496 of the 517 filtered-DEGs were upregulated both in REV vs. FLO and REV vs. INF, suggesting the filtered-DEGs likely promoted the induction of flower reversion (detailed gene lists are provided in Supplementary Table S2).

### Expression Pattern Verification Using qPCR

To verify the efficacy of filtered-DEGs, we performed qPCR with 12 of the high-expression filtered-DEGs (Supplementary Presentation S2). The results demonstrated that all examined genes showed expression trends similar to the RNA-seq results, with variation in the relative expression levels of some genes (Table 2). Upregulation of the genes 3NG053500, 5NG047700, and J517000 was more prominent in qPCR, while with the genes 1KG404800 and 1KG545400, it was less prominent. The difference might be caused by different qPCR and RNA-seq measurements of expression levels (Wagner et al., 2012; Biassoni and Raso, 2014) and differences in amplification efficiency of the primers.

**Table 2:** Real-time RT-PCR analysis of 12 predicted-DEGs.

Gene_ID	Ref_Gene_Name	REV vs. INF		REV vs. FLO	
		qPCR(ΔΔCt)	Log_2_(FC)	qPCR(ΔΔCt)	Log_2_(FC)
MSTRG.2904	1KG404800	13.37 ± 1.40	6.0414	1.41 ± 0.09	7.7163
MSTRG.3995	1KG545400	0.74 ± 0.69	2.65	2.87 ± 0.14	3.6034
MSTRG.11392	2KG439000	7.88 ± 1.45	2.8805	4.20 ± 0.36	2.6978
MSTRG.16830	2NG586800	8.27 ± 1.20	1.6391	0.86 ± 0.49	2.9134
MSTRG.17552	3KG025800	5.72 ± 1.80	1.8881	3.49 ± 0.07	2.5684
MSTRG.22101	3NG053500	9.98 ± 0.57	4.9213	9.80 ± 0.62	4.2443
MSTRG.35624	5NG047700	13.93 ± 1.28	5.1465	13.05 ± 1.41	5.2529
MSTRG.49482	7NG099700	2.30 ± 0.62	3.0658	2.89 ± 1.13	3.4403
MSTRG.51245	7NG306800	8.24 ± 1.08	4.8722	5.20 ± 1.46	5.2849
MSTRG.54457	8KG390300	3.04 ± 1.94	3.3022	2.09 ± 0.58	3.5506
MSTRG.55330	8NG161900	3.68 ± 1.50	3.8521	4.15 ± 0.29	4.563
MSTRG.72402	J517000	13.60 ± 0.75	3.0249	9.02 ± 0.58	3.4384

*REV, material obtained by floral reverse induction on SIM medium; FLO, material obtain by in vitro flower induction on MS medium; INF, immature inflorescence without incubation. ΔΔCt, relative gene expression by qRT-PCR; Log_2_(FC), expression difference analysis of RNA-seq. Three replications were included in qPCR analysis.*

### Gene Ontology Classification of Filtered-DEGs

**FIGURE 4:**
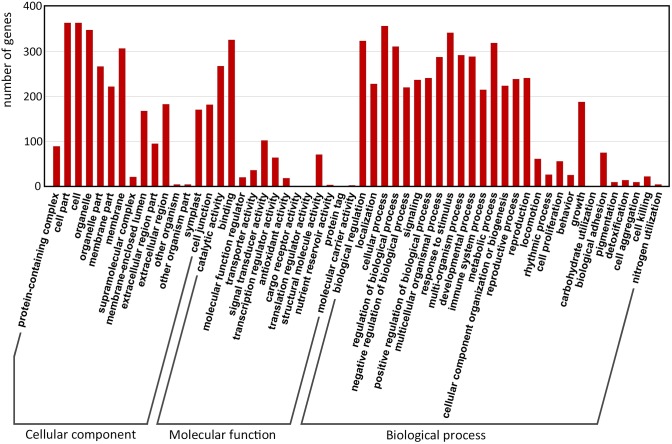
Gene Ontology classification of predicted-DEGs. A total of 380 DEGs were annotated by at least one of the three categories: biological process, cellular component, and molecular function. The *x*-axis indicates the level 2 GO terms, and the *y*-axis means the number of genes.

Gene Ontology (GO) enrichment of the filtered-DEGs provided insight into the function of target genes in plant developmental processes. Overall, 380 of 517 (67.7%) filtered-DEGs were annotated by at least one of the three categories of the GO function classification. Among these filtered-DEGs, 372 genes were related to cellular components, 356 genes were involved in molecular function, and 376 genes were involved in biological processes (Figure 4).

Within ‘cellular components,’ the most enriched filtered-DEGs were ‘cell,’ ‘cell part,’ ‘organelle,’ and ‘membrane.’ Within ‘molecular function,’ the most enriched items were ‘binding,’ ‘catalytic activity,’ ‘signal transducer activity,’ ‘structural molecule activity,’ and ‘transcription regulator activity’ terms. Within ‘biological process’, the most enriched items were ‘cellular process,’ ‘response to stimulus,’ ‘biological regulation,’ ‘metabolic process,’ and ‘regulation of biological process’ (Figure 4). Furthermore, the GO terms ‘reproduction,’ ‘reproductive process,’ ‘signaling,’ and ‘growth’ were also greatly enriched. These results suggest that signal transduction is essential for switchgrass flower reversion, and that most of the biological processes involved in switchgrass flower reversion are related to the cell membrane.

### COG Classification of Predicted-DEGs

**FIGURE 5:**
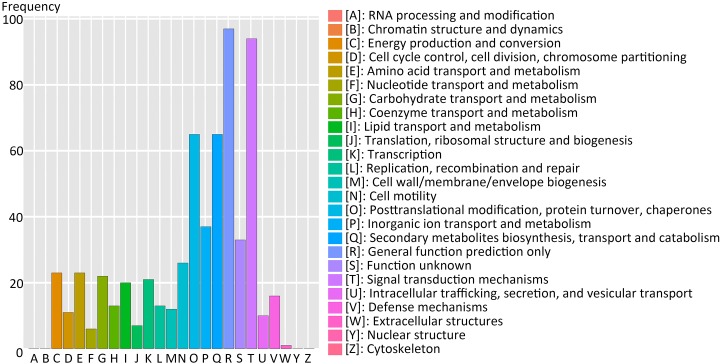
Cluster of Orthologous Groups database of proteins (COG) functional classification of the predicted-DEGs. A total of 214 DEGs were annotated and assigned to 21 functional clusters. The *x*-axis indicates the COG categories as listed on the right, and *y*-axis means the number of genes.

Using the Clusters of Orthologous Groups (COG) database of proteins, 214 genes of 517 (41.3%) filtered-DEGs were annotated and assigned to 21 functional clusters (Figure 5). The highest annotation frequency was ‘general function prediction only’ (97 genes, 45.3%), followed by ‘signal transduction mechanisms’ (94 genes, 43.9%), ‘posttranslational modification, protein turnover, and chaperones’ (65 genes, 30.4%), ‘secondary metabolites biosynthesis, transport and catabolism’ (65 genes, 30.4%), and ‘inorganic ion transport and metabolism’ (37 genes, 17.3%). We can deduce that signal transduction-related genes are essential for switchgrass flower reversion. Furthermore, there were 10 and 21 genes upregulated in functional clusters of ‘cell cycle control, cell division, and chromosome partitioning’ and ‘transcription,’ respectively.

### Transcription Factors Implicated in Switchgrass Floral Reversion

Transcription factors (TFs) are a group of genes with different DNA-binding domains that influence the stress response and developmental processes in plants. We analyzed predicted-DEGs using a TF prediction tool^2^ (Jin et al., 2017), and 37 genes were annotated. Of these genes, 18 were assigned to the WRKY family, seven were assigned to the C2H2 family, seven were assigned to the NAC family, and each of the remaining five were assigned to bHLH, ERF, HSF, MYB, and TCP families. The WRKY TF family has the largest number of DEGs, which suggested that they play important roles in switchgrass flower reversion. Analysis showed that all 37 genes were upregulated (Supplementary Table S3).

### KEGG Pathway Annotation of Predicted-DEGs

Pathway-based KEGG analysis of filtered-DEGs annotated 106 (20.5%) genes, of which 72 were enriched by 65 pathways. The most enriched pathway was ‘phenylpropanoid biosynthesis’ (12 genes, 2.3%), followed by ‘plant hormone signal transduction’ (9 genes, 1.7%), ‘plant–pathogen interaction’ (8 genes, 1.5%), and ‘MAPK signaling pathway - plant’ (6 genes, 1.1%) (Supplementary Table S4).

Of the 72 enriched filtered-DEGs, 15 genes were functionally related to plant hormone biosynthesis or signal transduction (Table 3). In the ‘zeatin biosynthesis’ and ‘plant hormone signal transduction’ pathways, six genes related to cytokinin biosynthesis or signal transduction were enriched (Table 3). We added 3 mg.L^-1^ benzylaminopurine (BAP) to the medium for switchgrass flower reversion induction. Genes involved in zeatin biosynthesis were annotated as *cytokinin dehydrogenase* (*CKX*, Pavir.5NG047700 and Pavir.J496300) and *cis-zeatin O-glucosyltransferase* (*CISZOG*, Pavir.7KG284900 and Pavir.7KG285000) respectively, which function in cytokinin degradation or *cis*-zeatin *O*-glucosylation, instead of as promoters of zeatin biosynthesis. Pavir.2KG175300 was annotated as a cytokinin receptor located at the membrane (Yamada et al., 2001) and Pavir.3NG010100 was annotated as a type-A *Arabidopsis* response regulator downstream of the cytokinin signal response (Hwang et al., 2012), and both were upregulated in reverse-developing explants. Three filtered-DEGs were annotated as auxin related genes (Table 3), of which, Pavir.2NG493200 was annotated as catalyzing indole-3-acetamide to indole-3-acetate (IAA) (Pollmann et al., 2003), and Pavir.3NG328700 and Pavir.4KG369800 were annotated as positive effectors of cell expansion downstream of the auxin signaling pathway (Spartz et al., 2012). In ethylene biosynthesis and signaling-related pathways, two genes were enriched (Table 3). Pavir.7NG301900 was annotated as catalyzing *S*-adenosyl-L-methionine to 1-aminocyclopropane-1-carboxylate, which is required for ethylene synthesis (Arteca and Arteca, 1999), and Pavir.9NG018300 was annotated as an ethylene response factor 1 (*ERF1*) TF involved in ethylene signal transduction. Additionally, two genes (Pavir.2NG586800 and Pavir.9KG267400) were annotated as *Jasmonate ZIM-domain* (*JAZ*) proteins and were involved in JA signaling (Yu et al., 2018), one gene was annotated as *NPR1* and was involved in salicylic acid signaling, and one gene was annotated as *SNRK2* and was involved in ABA signaling (Table 3, Fujii and Zhu, 2009).

**Table 3:** Kyoto Encyclopedia of Genes and Genomes (KEGG) enriched DEGs associated with hormone biosynthesis and signaling.

Gene_ID	Ref_Gene_ Name	Regulation	KEGG annotation	Related function
MSTRG.35624	5NG047700	Up	K00279, cytokinin dehydrogenase	Zeatin biosynthesis
MSTRG.72247	J496300	Up	K00279, cytokinin dehydrogenase	Zeatin biosynthesis
MSTRG.47642	7KG284900	Up	K13495, *cis*-zeatin O-glucosyltransferase	Zeatin biosynthesis
MSTRG.47643	7KG285000	Up	K13495, *cis*-zeatin O-glucosyltransferase	Zeatin biosynthesis
MSTRG.9518	2KG175300	Up	K14489, arabidopsis histidine kinase 2/3/4 (cytokinin receptor)	Cytokinin signaling
MSTRG.21760	3NG010100	Up	K14492, two-component response regulator ARR-A family	Cytokinin signaling
MSTRG.16127	2NG493200	Up	K01426, amidase	Indole-3-acetate biosynthesis
MSTRG.23917	3NG328700	Up	K14488, SAUR family protein	Auxin signaling
MSTRG.26615	4KG369800	Up	K14488, SAUR family protein	Auxin signaling
MSTRG.51213	7NG301900	Up	K20772, 1-aminocyclopropane-1-carboxylate synthase 1/2/6	Ethylene biosynthesis
MSTRG.61817	9NG018300	Up	K14516, ethylene-responsive transcription factor 1	Ethylene signaling
MSTRG.16830	2NG586800	Up	K13464, jasmonate ZIM domain-containing protein	Jasmonic acid signaling
MSTRG.58502	9KG267400	Up	K13464, jasmonate ZIM domain-containing protein	Jasmonic acid signaling
MSTRG.63047	9NG170200	Up	K14508, regulatory protein NPR1	Salicylic acid signaling
MSTRG.59586	9KG401100	Down	K14498, serine/threonine-protein kinase SRK2	Abscisic acid signaling

### Gene Function Investigation of Filtered-DEGs

**FIGURE 6:**
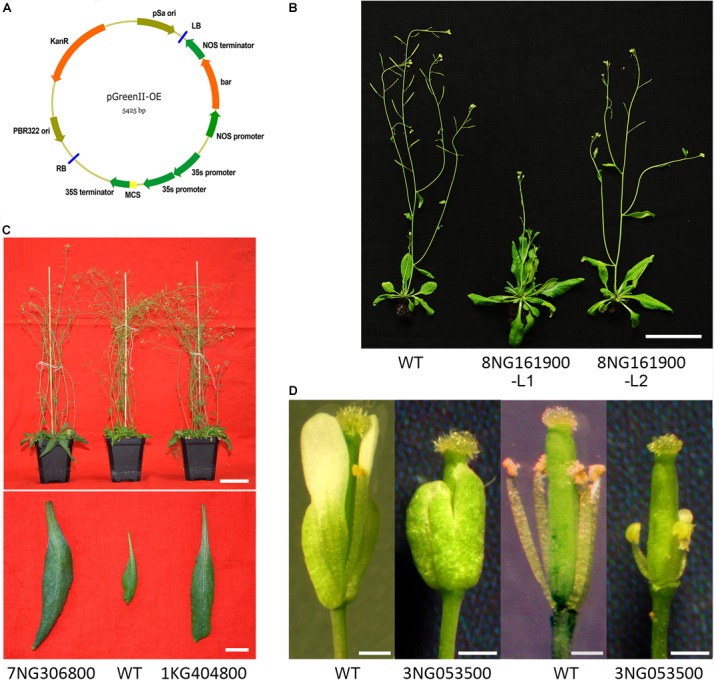
Investigation of the gene function of filtered-DEGs. **(A)** Expression vector employed. **(B)** Transgenic Pavir.8NG161900 plants compared with wild-type *Arabidopsis*. **(C)** Transgenic Pavir.7NG306800 and Pavir.1KG404800 plants compared with wild-type *Arabidopsis*. **(D)** Flowers of transgenic plants of Pavir.3NG053500 compared with wild-type *Arabidopsis*.

To verify the function of the filtered-DEGs in flower reversion, we selected four filtered-DEGs with high expression and large expression alteration refer to qPCR and RNA-seq results. The open reading frames were cloned into the pGreenII-based expression vector (Figure 6A and Supplementary Table S5; Hellens et al., 2000). Genes were transformed into the wild-type Colombia *Arabidopsis* by an *Agrobacterium*-mediated floral dip method (Clough and Bent, 1998). Gene transformation was verified by genotyping, gene expression was examined by semi-qPCR (Supplementary Presentation S1), and the phenotypes of screened offspring were further analyzed.

Ectopic expression of gene Pavir.8NG161900 (*cysteine-rich RLK*) caused delayed flowering time in *Arabidopsis* compared with wild-type plants (Figure 6B). Moreover, rosette and cauline leaves were enlarged, although the leaf number was unchanged (Figure 6B). Overexpression of Pavir.3NG053500 (*dirigent protein*) led to extremely shortened stamen filaments and undeveloped petals (Figure 6D). Furthermore, the transgenic plant was sterile and no seeds were produced when crossed and reciprocal crossed with wild-type plants. In transgenic Pavir.7NG306800 (*F3H-2*) and Pavir.1KG404800 (*putative ripening-related protein 2*) *Arabidopsis* plants, neither flowering structure nor timing was changed (Kim et al., 2008). However, the flower number was largely reduced (Figure 6C) and rosette leaves were enlarged (Figure 6C) compared to wild-type plants.

## Discussion

While flower reversion is an abnormal developmental process in plants, it allows the study of flower development from an interesting perspective. In this study, INFs from the switchgrass cultivar ‘Alamo’ incubated on SIM medium showed flower reversion, while those incubated on MS medium produced *in vitro* flowers. Anatomical and histological analyses showed that the emerged shoots originated from flower reversion and initiated from the pistil primordia in the floret primordium. Pairwise gene expression comparisons among REV, FLO, and INF generated 517 DEGs considered to regulate switchgrass flower reversion.

Cytokinin is an important plant hormone that regulates plant growth and development, and is considered to promote flower development. In *Arabidopsis*, exogenously applied BAP promoted flower formation in wild-type Columbia grown plants under 8 h short days (D’Aloia et al., 2011). In rice (*Oryza sativa*), *LONELY GUY* (*LOG*) encodes a cytokinin-activating enzyme and *GRAIN NUMBER1* (*GN1*) encodes a *cytokinin oxidase* (*CKX2*), the *log* mutations caused small meristems and the *gn1* mutations generated large inflorescence meristems and more grains (Yoshida and Nagato, 2011). In oilseed rape (*Brassica napus*), the content of all isoprenoid cytokinins increased significantly during vernalization, an essential process for inflorescence formation (Tarkowská et al., 2012). However, cytokinin can inhibit floral organ development. Overexpression of a cytokinin synthase *isopentenyltransferase 4* (*AtIPT4*) in *Arabidopsis* increased cytokinin levels, and flowers of the transgene plants were abnormally developed (Li et al., 2010). *AP1* is an important gene for the establishment of floral meristems, and functions as a suppressor of *LOG* and activator of the cytokinin degradation gene *cytokinin oxidase 3* (*CKX3*). This suggests that plants will avoid cytokinin accumulation during flower meristem formation (Han et al., 2014). In the present study, exogenous application of BAP induced flower reversion in switchgrass. Gene expression analysis showed that two genes (Pavir.2KG175300 and Pavir.3NG010100) involved in cytokinin signal transduction were upregulated in REV. Four genes (Pavir.5NG047700, Pavir.J496300, Pavir.7KG284900, and Pavir.7KG285000) that degrade or inactivate cytokinin were also upregulated in REV, possibly due to feedback regulation from high concentrations of the exogenously applied cytokinin analog.

Additionally, the expression levels of genes related to biosynthesis and signal transduction of other hormones were also affected. Ethylene is an important hormone in plant reproductive development and fruit ripening, but may also play a role in inhibiting stamens and promoting carpel development in the early stage of melon (*Cucumis melo*) flower formation (Switzenberg et al., 2014). In switchgrass, genes associated with ethylene biosynthesis (Pavir.7NG301900) and signal transduction-related gene (Pavir.9NG018300) were upregulated in REV explants, suggesting that ethylene-related genes are involved in inhibiting flower development in switchgrass flower reversion. Besides, high levels of ethylene increased *SAUR* expression, a downstream auxin response gene, in *Arabidopsis* (Markakis et al., 2013). Two *SAUR* genes and an IAA biosynthesis-related gene were also upregulated in REV explants. JA is a widely studied hormone involved in the plant response to biotic and abiotic stressors, and reportedly contributes to rice spikelet morphogenesis by preventing *OsMYC2* from activating *OsMADS1*, an E-class gene crucial for spikelet development (Cai et al., 2014). Two orthologs of *JAZ* in switchgrass were upregulated in REV explants, suggesting they function as flower development inhibitors in the reversion process. In addition, the expression of salicylic acid and ABA signaling-related genes was also altered. To conclude, plant hormone regulation networks were influenced by the addition of BAP in REV, and the reconstruction of plant hormone biosynthesis and signal transduction affected switchgrass flower reversion.

As mentioned above, our understanding of flower reversion is based on the analysis of relevant mutants (*AGL24*, *LFY*, and *AG* in *A*. *thaliana*; *TM29* and *SFT* in *S*. *lycopersicum*; *FBP2* in *Petunia hybrid*; *IFA1*, *ID1*, *ZFL1*, and *ZFL2* in *Z*. *mays*; Laudencia-Chingcuanco and Hake, 2002; Molinero-Rosales et al., 2004). However, flower reversion is not simply a process of reversed flower development; genes that distinctively function in flower reversion were not detected in flower organ mutant studies. Comparison of flower reversion-related genes reported in previous research and the 517 DEGs obtained in this study revealed no overlap, further illustrating the difference between flower reversion and flower development.

Switchgrass flower reversion is a complex process. We examined functions of filtered-DEGs, four of which were overexpressed in *Arabidopsis*. Among them, Pavir.8NG161900 and Pavir.3NG053500 inhibited reproductive development, while Pavir.7NG306800 and Pavir.1KG404800 promoted vegetative development. No plant was observed with complete flower reversion in any transgene event; this might be attributed to the complexity of flower reversion in switchgrass, which is regulated by multiple genes (517 possible related genes) and the overexpression of a single gene is insufficient to induce the complete process of flower reversion. Nevertheless, flower reversion involves multiple biological processes, including the promotion of vegetable growth, suppression of flower formation, and dedifferentiation of flower organs. Although overexpression of the filtered-DEGs did not show complete flower reversion, vegetative and reproductive growth were affected, both of which are important in the flower reversion process.

## Conclusion

Using the Illumina platform, we analyzed the gene expression of immature switchgrass inflorescences incubated on SIM and MS media, and uncultured INFs. Comparison of gene expression patterns identified 517 genes involved in switchgrass floral reversion. Based on the annotations of the NR, Swiss-Prot, GO, COG, and KEGG databases, we found that signal transduction and metabolism are essential for switchgrass flower reversion. Switchgrass flower reversion is a complex process that involves multiple biological processes, including the promotion of vegetative growth, suppression of flower formation, and dedifferentiation of flower organs; this was supported by the overexpression phenotypes of some of the filtered-DEGs in *Arabidopsis*.

## Materials and Methods

### Plant Materials and Explant Culture

The lowland switchgrass cultivar ‘Alamo’ was used in this study. Plants were grown in an outdoor field located at the Northwest A&F University in Yangling, Shaanxi, China (108.072°E, 34.295°N). Shoot apexes were harvested at the E4 stage in early July (Moore et al., 1991). The shoot apexes were surface sterilized with 70% ethanol and 8% sodium hypochlorite. After rinsing three times with sterile distilled water, shoot apexes were then cut by 0.5 cm at both ends and split longitudinally. Both halves of the shoot apex were placed, section downward, on solid MS (Murashige and Skoog, 1962) or SIM (Chai et al., 2012) medium and incubated under constant temperature (25°C) and a 20 h light/4 h dark photoperiod. The MS medium consisted of MS salts, organics, vitamins, 7.5 g/L^-1^ agar, and 30 g/L^-1^ sucrose. The SIM medium was MS medium with 3 mg/L^-1^ benzylaminopurine (BAP, Sanland, Fujian, China). More than 90% of the explants on the SIM medium induced neonatal shoots from flower reversion after 60 days of incubation.

### Microscopic Structure Analysis of Switchgrass Floral Reversion

Shoot clumps produced on SIM medium were harvested and dismembered under a stereoscopic microscope (Nikon SMZ1500, Japan), and their morphological and anatomical characteristics were identified and described. Ten explants were analyzed per treatment.

Separated shoots were fixed with FAA (formalin: acetic acid: 70% alcohol = 5:5:90) stationary liquid for 6 h and stained with hematoxylin (Maikun, Shanghai, China) for 3 days, followed by color differentiation in tap water for 1 h (Avwioro, 2011). Stained materials were dehydrated with a gradient alcohol series (20 min each of 70, 80, and 90%, followed by 100% twice for 15 min each), cleared with xylene, and infiltrated with melted paraffin wax. Then, they were embedded in a paraffin block and cut into 10 μm slices using a paraffin microtome (JinHuaHuiYou HY-202A, China). Slices were dewaxed with xylene, post-mounted with Permount Mounting Medium (HuShi, Shanghai, China), and observed under an optical microscope (Chongqing UOP, UB203i, China).

### RNA Isolation and mRNA-seq

The explants incubated for 20 days on MS and SIM media were flash frozen in liquid nitrogen together with uncultured INFs. Four replications were included in each treatment, and five explants were employed for each replicate.

Total RNA was isolated using TRIzol reagent (Invitrogen, Carlsbad, CA, United States) according to the manufacturer’s instructions. RNA quality and quantity were determined using electrophoresis on a 1.5% agarose gel using an Agilent 2100 Bioanalyzer (Agilent Technologies, Santa Clara, CA, United States) and a Nanodrop 2000 Spectrophotometer (Thermo Fisher Scientific, Waltham, MA, United States), respectively. mRNA was purified using a NEBNext Poly(A) mRNA Magnetic Isolation Module (E7490; New England Biolabs, Ipswich, MA, United States). The sequencing library for each sample was constructed using the NEBNext mRNA Library Prep Master Mix Set for Illumina (E6110; New England Biolabs) and the NEBNext Multiplex Oligos for Illumina (E7500; New England Biolabs). The insertion length was set to 180 bp and verified by electrophoresis on a 1.8% agarose gel. After quantifying the sequencing libraries with Library Quantification Kit-Illumina GA Universal (KK4824; Kapa Biosystems, Inc., Wilmington, MA, United States), the libraries were used for cluster station generation on Illumina cBot (Illumina, San Diego, CA, United States). Paired-end sequencing was performed on an Illumina HiSeq 2500 (Illumina).

### Gene Expression Analysis and Mining of DEGs

Paired-end reads with a length of 202 bp were assessed with Q20, Q30, and GC content. Reads containing >20% low quality bases (quality score < 20) or >5% N were filtered out before assembly. All filtered transcriptome datasets were mapped versus the switchgrass genomic assembly^3^ (version 4.1; Casler et al., 2011) with Hisat2 (Kim et al., 2015) software and assembled by the Stringtie software without the ‘-e’ option to predict novel transcripts (Pertea et al., 2015, 2016). The assembled transcripts were merged together under the direction of GFF annotation (version 4.1) using Stringtie’s merge function to create a uniform set of transcripts for all 12 samples. The mapped reads were assembled again using Stringtie software with the uniform set of transcripts as a direction, and the ‘-e’ and ‘-B’ options were used to restrict novel transcript prediction and generate input file for DEG analysis (Pertea et al., 2016). The fold change was calculated using the R package ‘Ballgown’ (Fu et al., 2018), and DEGs were filtered with *p*-value ≤ 0.01 and fold change ≥ 2 as restrictions.

### Annotation of DEGs

The software program BLAST (Altschul et al., 1997) was used to provide the most inclusive functional description of the assembled sequences with a threshold *E*-value ≤ 1e-5. Annotation information was based on four sources: (1) the NR protein database (Pruitt et al., 2007) from the NCBI, (2) Swiss-Prot (Apweiler et al., 2004) from the Universal Protein Resource (UniProt), (3) the high-level functions and biological system pathway database KEGG (Kanehisa et al., 2004), and (4) the Cluster of Orthologous Groups (COG) database of proteins (Tatusov et al., 2000). We also obtained the GO annotation of genes based on the NR annotation using the Blast2GO program with E-value ≤ 1e-6 (Conesa et al., 2005) and GO functional classification for all genes using WEGO software (Ye et al., 2006).

### Real-Time qPCR Analysis

Total RNAs of reversed, unreversed, and uncultured INFs (three replications for each treatment) were isolated as described above. Using the PrimeScript RT reagent kit (Takara, Kyoto, Japan), the first strand of cDNA was synthesized according to the attached protocol. Real-time qPCR was performed using SYBR Premix EX Taq II Kit (Takara) on a QuantStudio 3 Flex Real Time System (Thermo Fisher Scientific). The total reaction volume was 20 μl, and the primers used are listed in Supplementary Table S6. The PCR reaction was performed as follows: 10 min at 95°C and 40 cycles of 94°C for 20 s and 60°C for 30 s. Data generated was analyzed using the ΔΔC_t_ method (Biassoni and Raso, 2014). Switchgrass gene ef1-α was used as an internal control (Gimeno et al., 2014).

### Gene Transformation of *Arabidopsis*

A binary vector based on pGreenII with a double 35S promoter for gene expression and a bar gene for selection was used (Hellens et al., 2000). A recombinant vector was introduced into the *Agrobacterium tumefaciens* strain GV3101 harboring the help plasmid pSoup and used for genetic transformation. *A*. *tumefaciens* was cultured at 28°C in liquid YEB medium with shaking at 250 rpm, and cells were harvested by centrifugation (5,000 rpm for 15 min at room temperature) and resuspended in infiltration medium to OD_600_ of approximately 0.8 (Clough and Bent, 1998). The gene transformation of *Arabidopsis* plants was performed according to the floral dip method (Clough and Bent, 1998) and seeds were harvested approximately 3–4 weeks later. After drying for 7 days at room temperature, seeds were sowed in well-watered soil and keep at 4°C for 2 days together with the plates, and the culture was transferred to normal conditions (Sanchez-Serrano and Salinas, 2014). After 7 days, seedlings with fully extended cotyledons were sprayed with 0.05% phosphinothricin (Sigma-Aldrich, St. Louis, MO, United States), and the surviving plants were further examined by PCR.

## Data Availability

The raw sequence reads are available at the NIH Short Read Archive database (accession number SRP144197).

## Author Contributions

XY and WY designed the experiments. WY, ZA, SF, LM, and XK conducted the experiments. ZC helped to conduct experiments. WY and LS wrote the manuscript. All authors read and approved the manuscript.

## Conflict of Interest Statement

The authors declare that the research was conducted in the absence of any commercial or financial relationships that could be construed as a potential conflict of interest.

## Footnotes

1 https://genome.jgi.doe.gov/portal/pages/dynamicOrganismDownload.jsf?organism=Pvirgatum

2 http://planttfdb.cbi.pku.edu.cn/prediction.php

3 https://genome.jgi.doe.gov/portal/pages/dynamicOrganismDownload.jsf?organism=Pvirgatum

## Abbreviations

ABA: abscisic acid
COG: Cluster of Orthologous Groups database of proteins
DEGs: differentially expressed genes
FLO: immature inflorescence produced floret on MS medium
GO: Gene Ontology
INF: immature inflorescence
JA: jasmonic acid
KEGG: Kyoto Encyclopedia of Genes and Genomes
MS: Murashige-Skoog
NCBI: National Center for Biotechnology Information
NR: non-redundant
REV: immature inflorescence produced flower reversion on SIM
RNA-seq: RNA sequencing
SIM: shoot induction medium
TF: transcription factor
