# Supplementary material for: Using Transcriptome Analysis to Identify Genes Involved in Switchgrass Flower Reversion
Source: Front Plant Sci. 2018 Dec 4;9:1805. doi: 10.3389/fpls.2018.01805 (PMC6288819; doi:10.3389/fpls.2018.01805)
Supplement: Supplementary file 7 [file Presentation_1.pptx]

## Slide 1
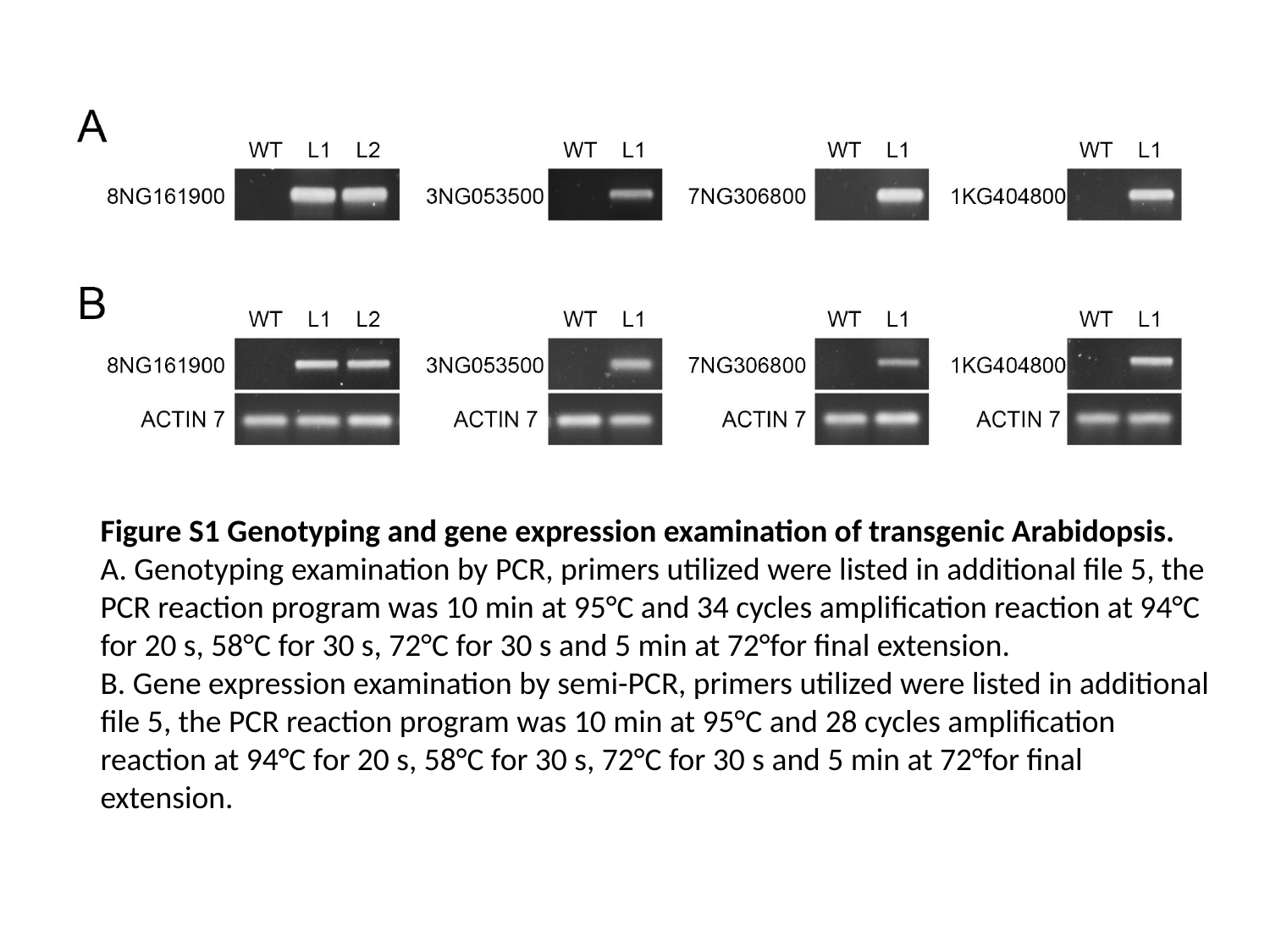

Figure S1 Genotyping and gene expression examination of transgenic Arabidopsis.
A. Genotyping examination by PCR, primers utilized were listed in additional file 5, the PCR reaction program was 10 min at 95°C and 34 cycles amplification reaction at 94°C for 20 s, 58°C for 30 s, 72°C for 30 s and 5 min at 72°for final extension.
B. Gene expression examination by semi-PCR, primers utilized were listed in additional file 5, the PCR reaction program was 10 min at 95°C and 28 cycles amplification reaction at 94°C for 20 s, 58°C for 30 s, 72°C for 30 s and 5 min at 72°for final extension.
