# Supplementary material for: Using Transcriptome Analysis to Identify Genes Involved in Switchgrass Flower Reversion
Source: Front Plant Sci. 2018 Dec 4;9:1805. doi: 10.3389/fpls.2018.01805 (PMC6288819; doi:10.3389/fpls.2018.01805)
Supplement: Supplementary file 8 [file Presentation_2.PPTX]

## Slide 1
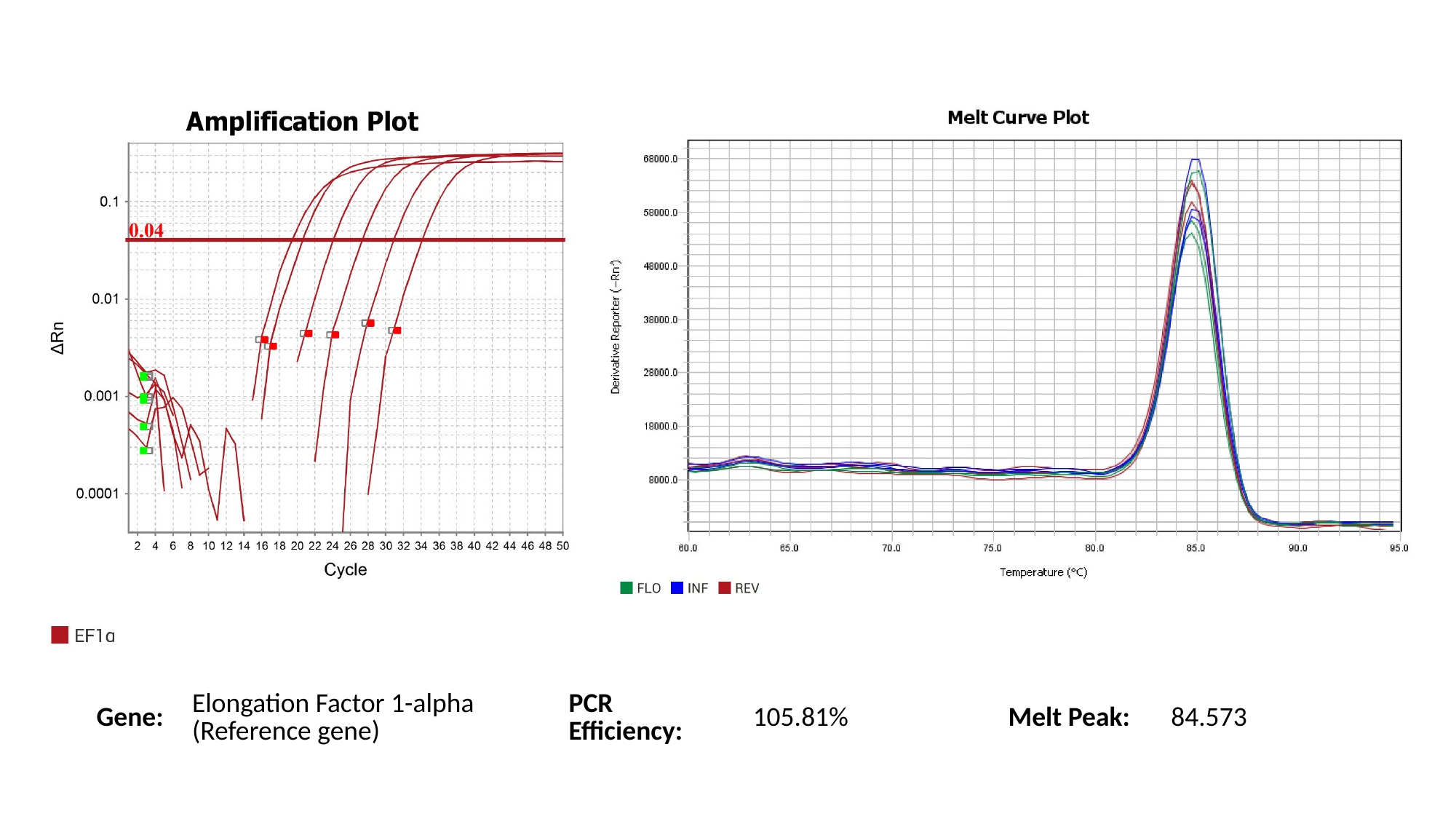

| Gene: | Elongation Factor 1-alpha (Reference gene) | PCR Efficiency: | 105.81% | Melt Peak: | 84.573 |
| --- | --- | --- | --- | --- | --- |

## Slide 2
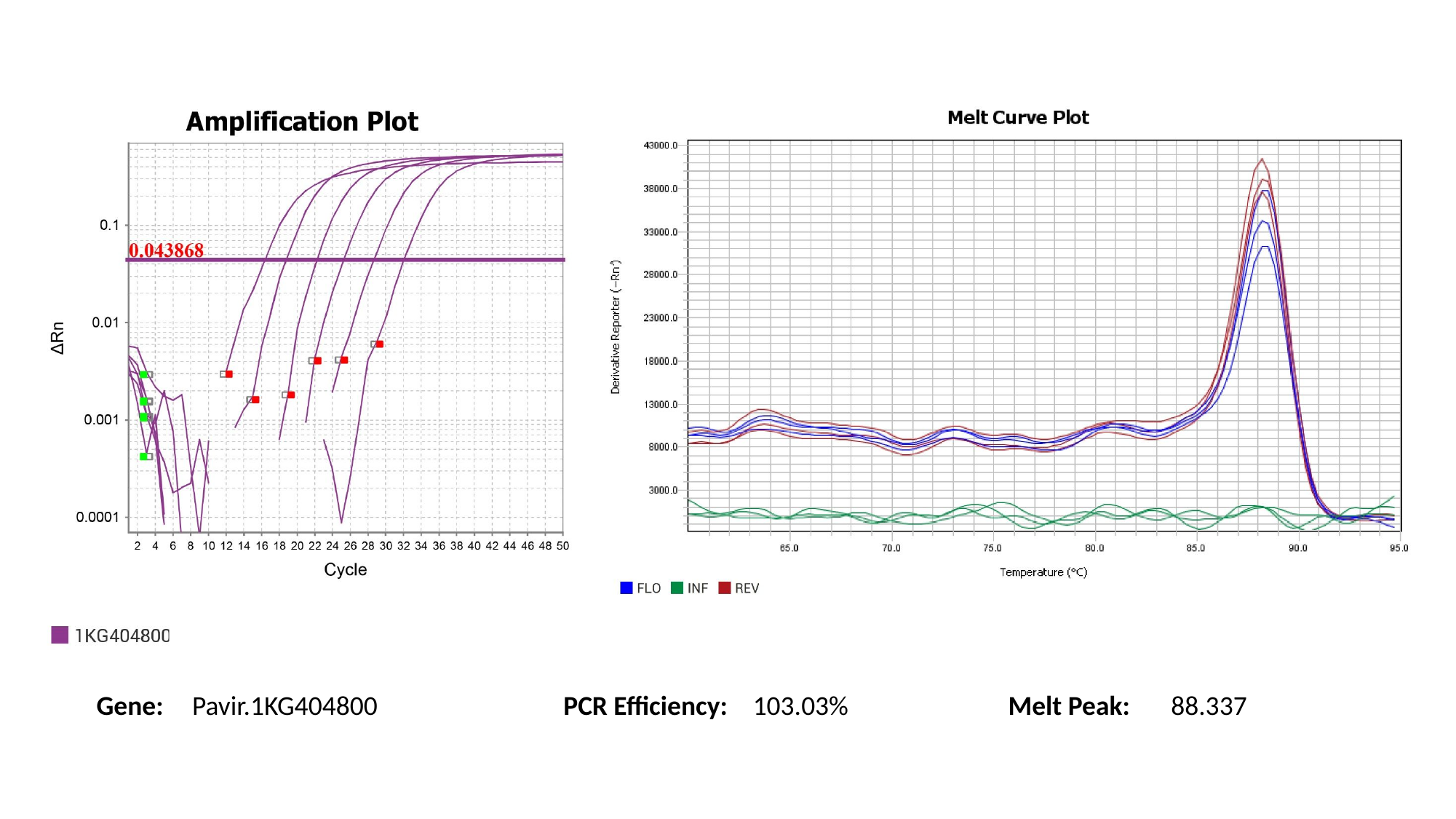

| Gene: | Pavir.1KG404800 | PCR Efficiency: | 103.03% | Melt Peak: | 88.337 |
| --- | --- | --- | --- | --- | --- |

## Slide 3
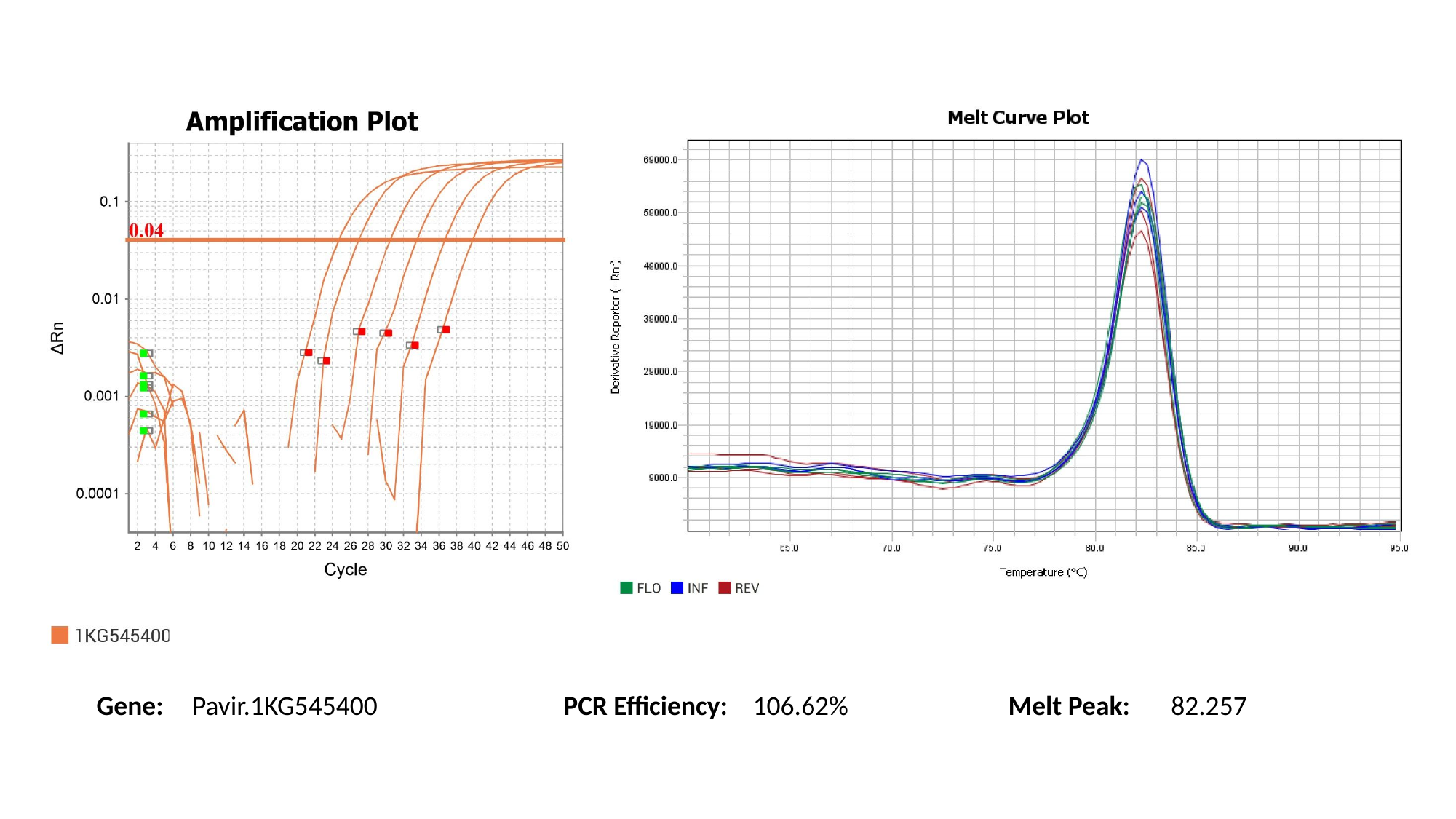

| Gene: | Pavir.1KG545400 | PCR Efficiency: | 106.62% | Melt Peak: | 82.257 |
| --- | --- | --- | --- | --- | --- |

## Slide 4
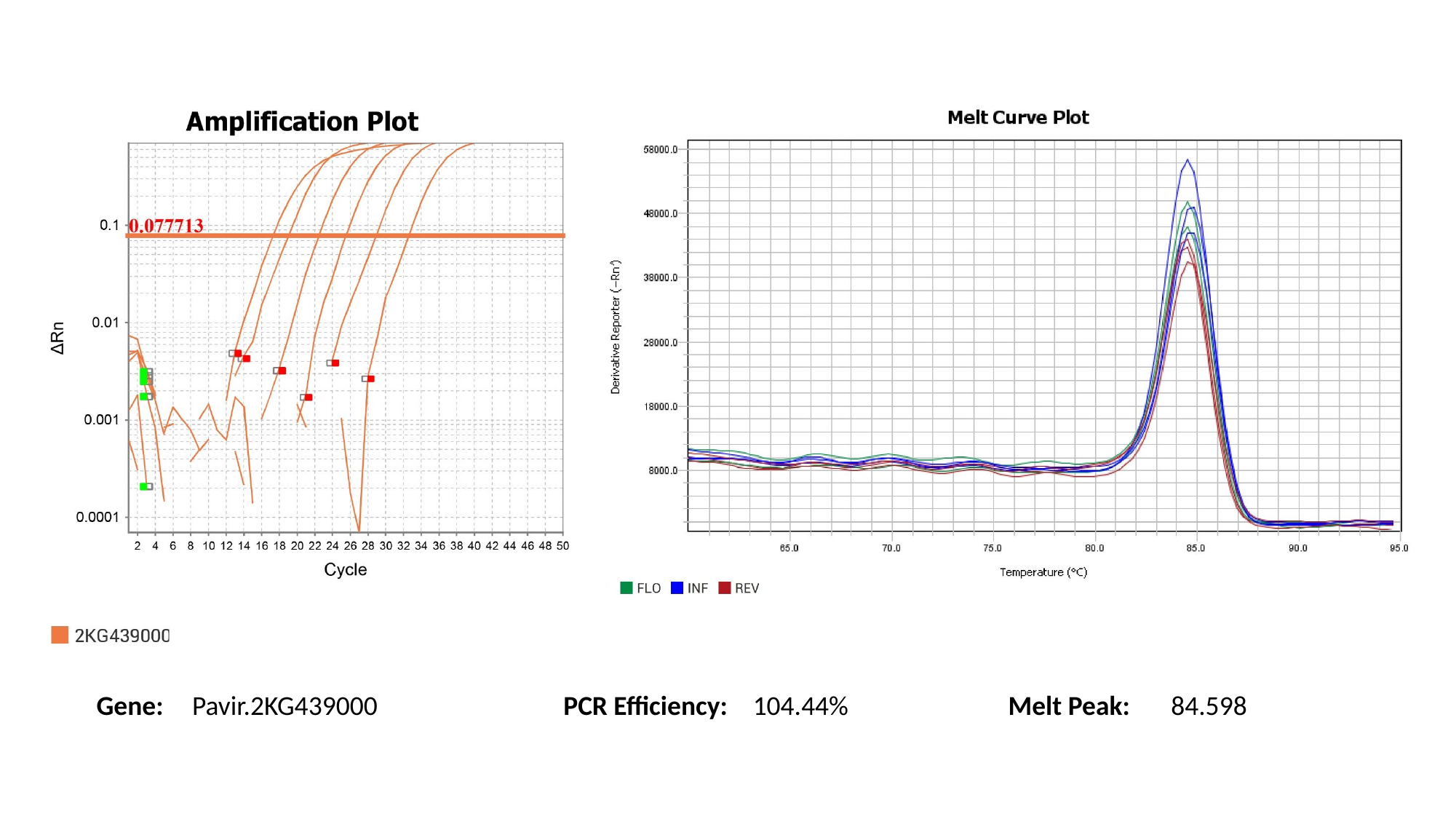

| Gene: | Pavir.2KG439000 | PCR Efficiency: | 104.44% | Melt Peak: | 84.598 |
| --- | --- | --- | --- | --- | --- |

## Slide 5
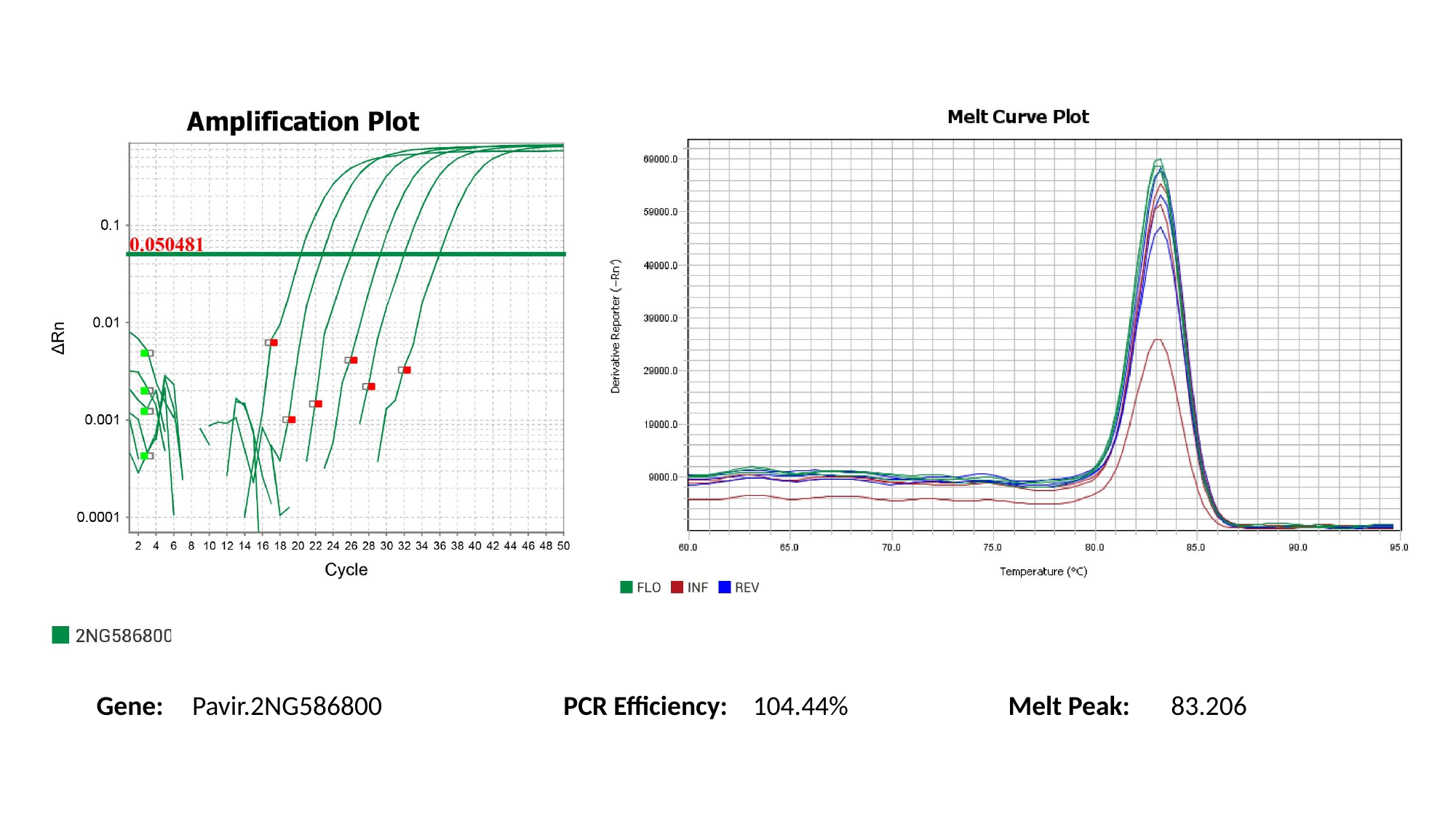

| Gene: | Pavir.2NG586800 | PCR Efficiency: | 104.44% | Melt Peak: | 83.206 |
| --- | --- | --- | --- | --- | --- |

## Slide 6
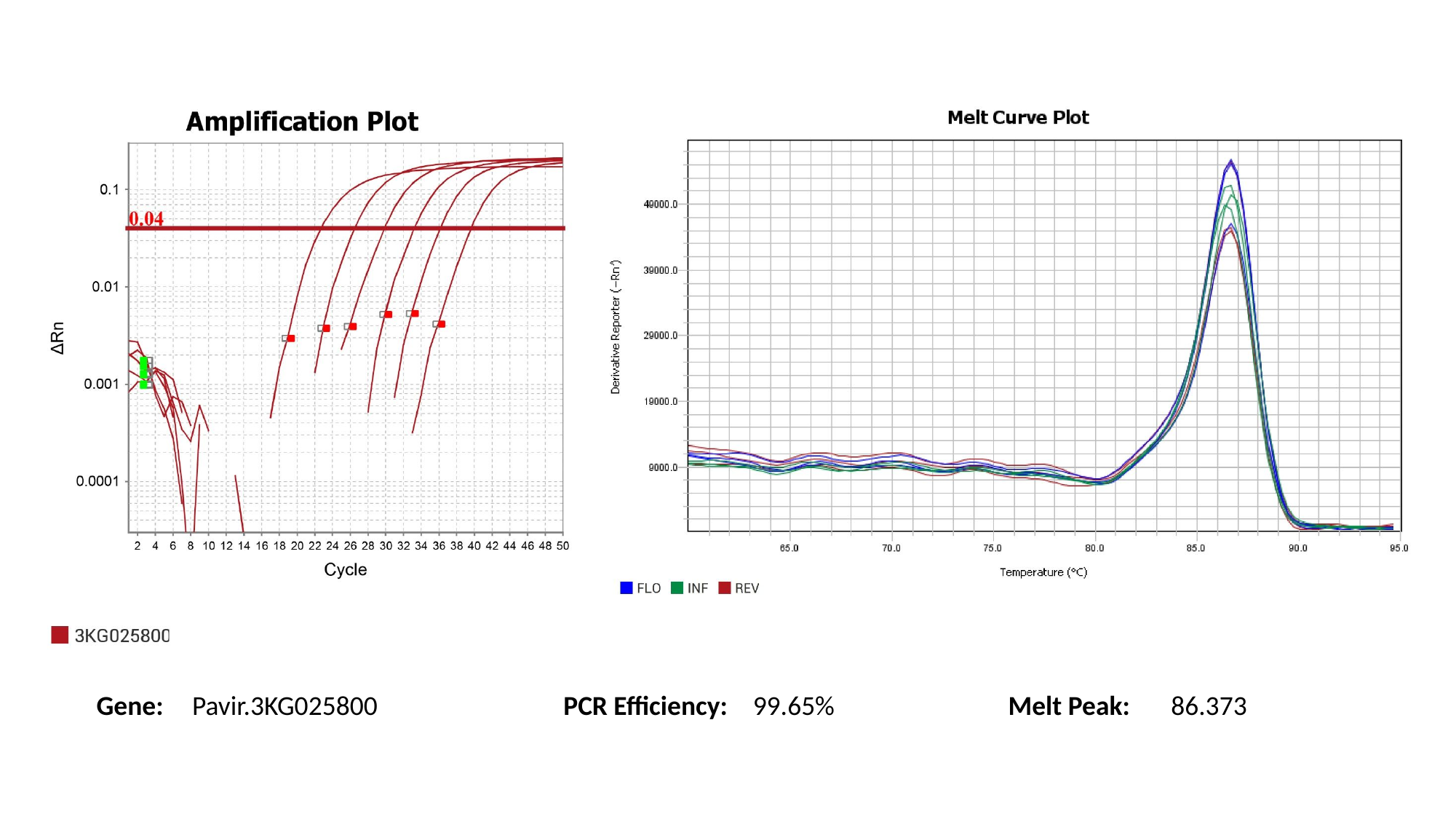

| Gene: | Pavir.3KG025800 | PCR Efficiency: | 99.65% | Melt Peak: | 86.373 |
| --- | --- | --- | --- | --- | --- |

## Slide 7
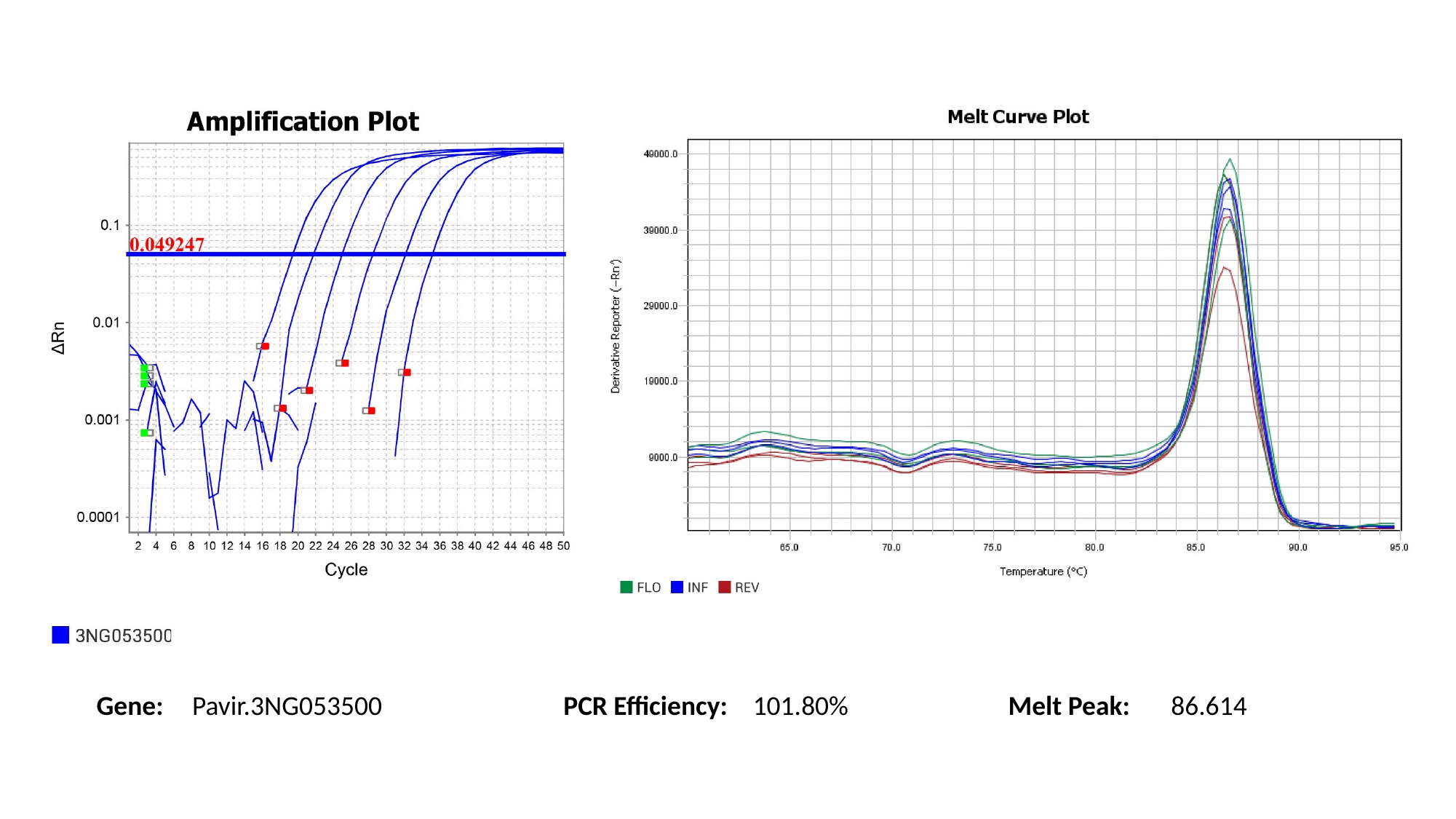

| Gene: | Pavir.3NG053500 | PCR Efficiency: | 101.80% | Melt Peak: | 86.614 |
| --- | --- | --- | --- | --- | --- |

## Slide 8
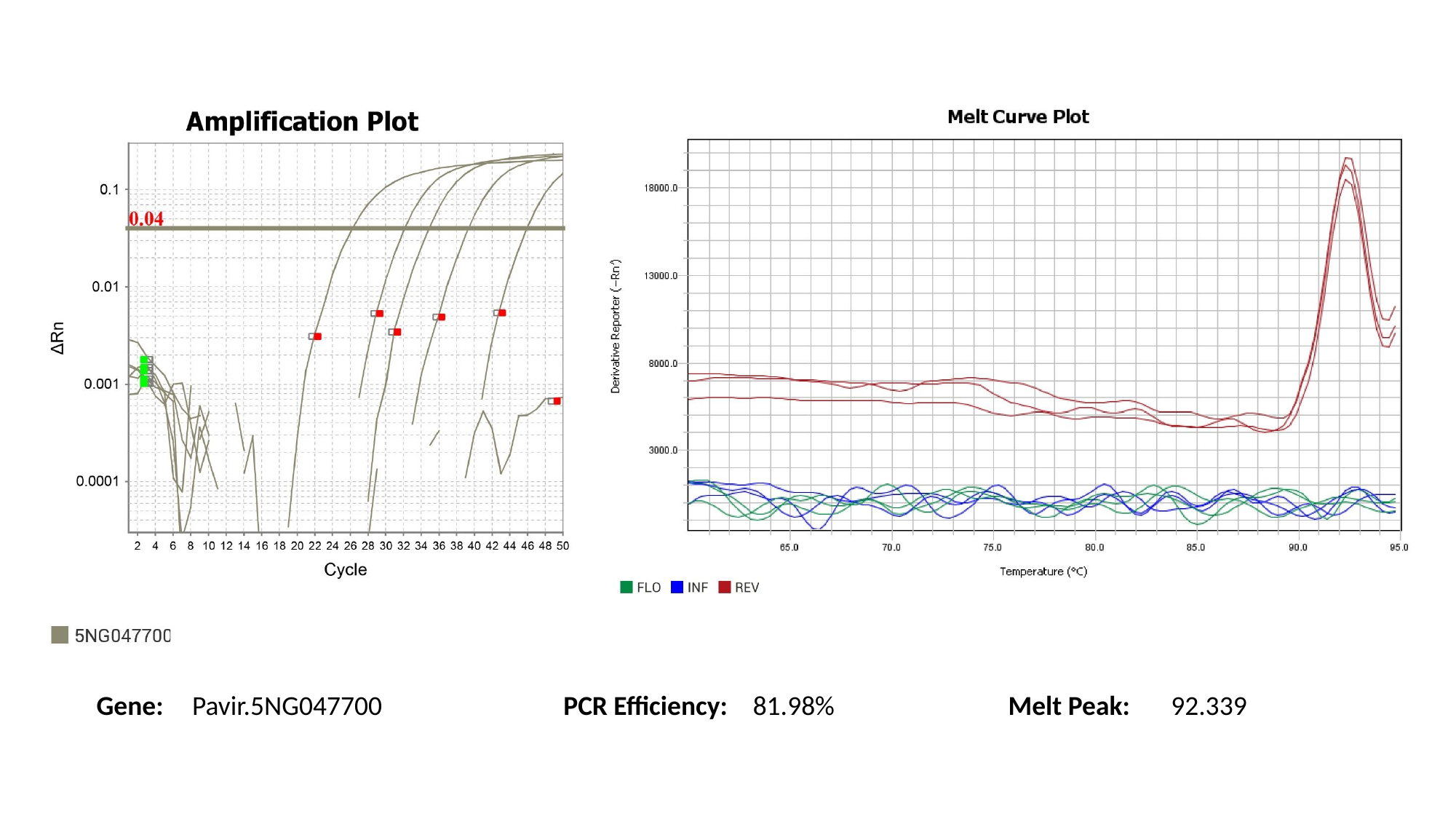

| Gene: | Pavir.5NG047700 | PCR Efficiency: | 81.98% | Melt Peak: | 92.339 |
| --- | --- | --- | --- | --- | --- |

## Slide 9
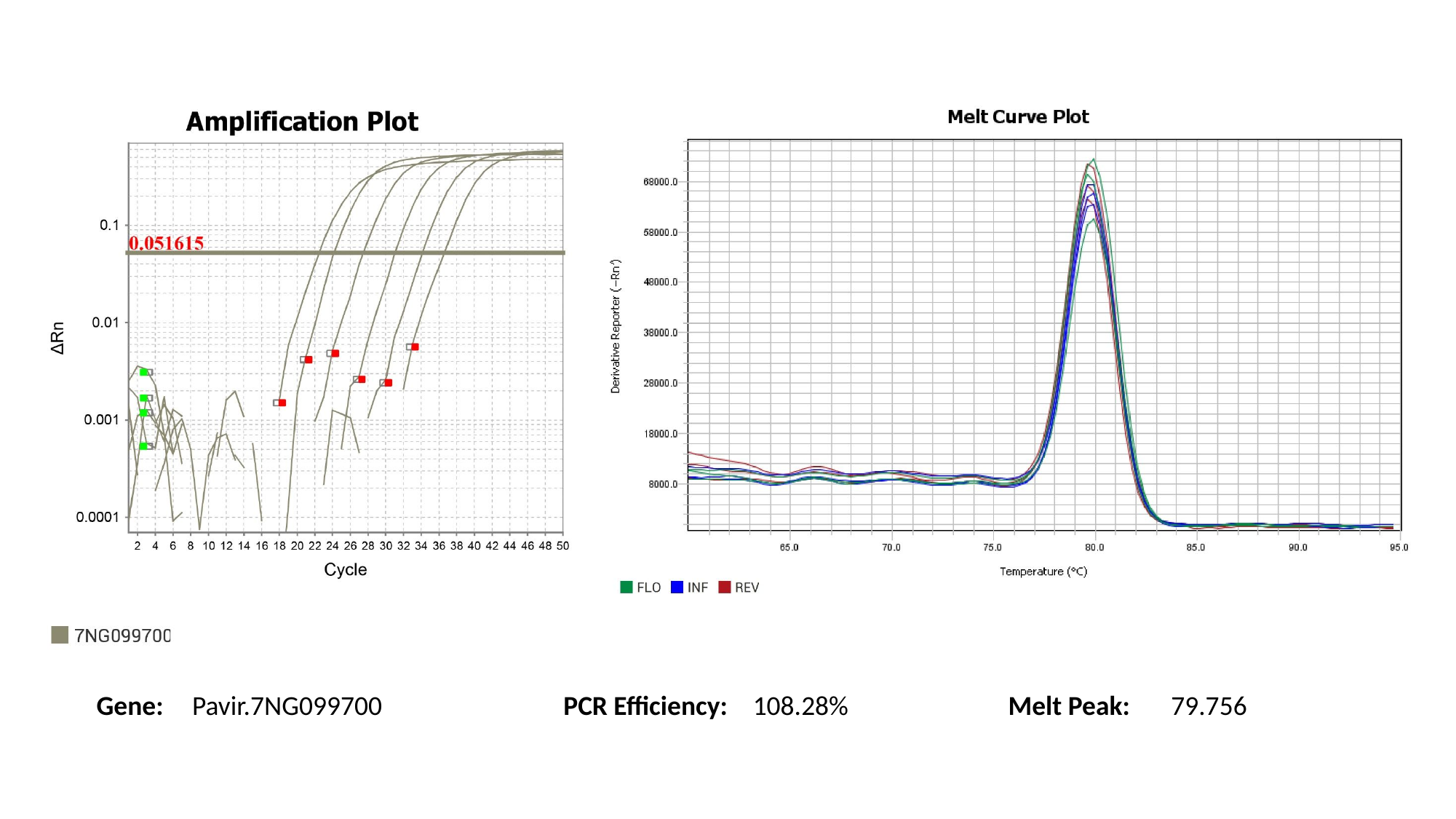

| Gene: | Pavir.7NG099700 | PCR Efficiency: | 108.28% | Melt Peak: | 79.756 |
| --- | --- | --- | --- | --- | --- |

## Slide 10
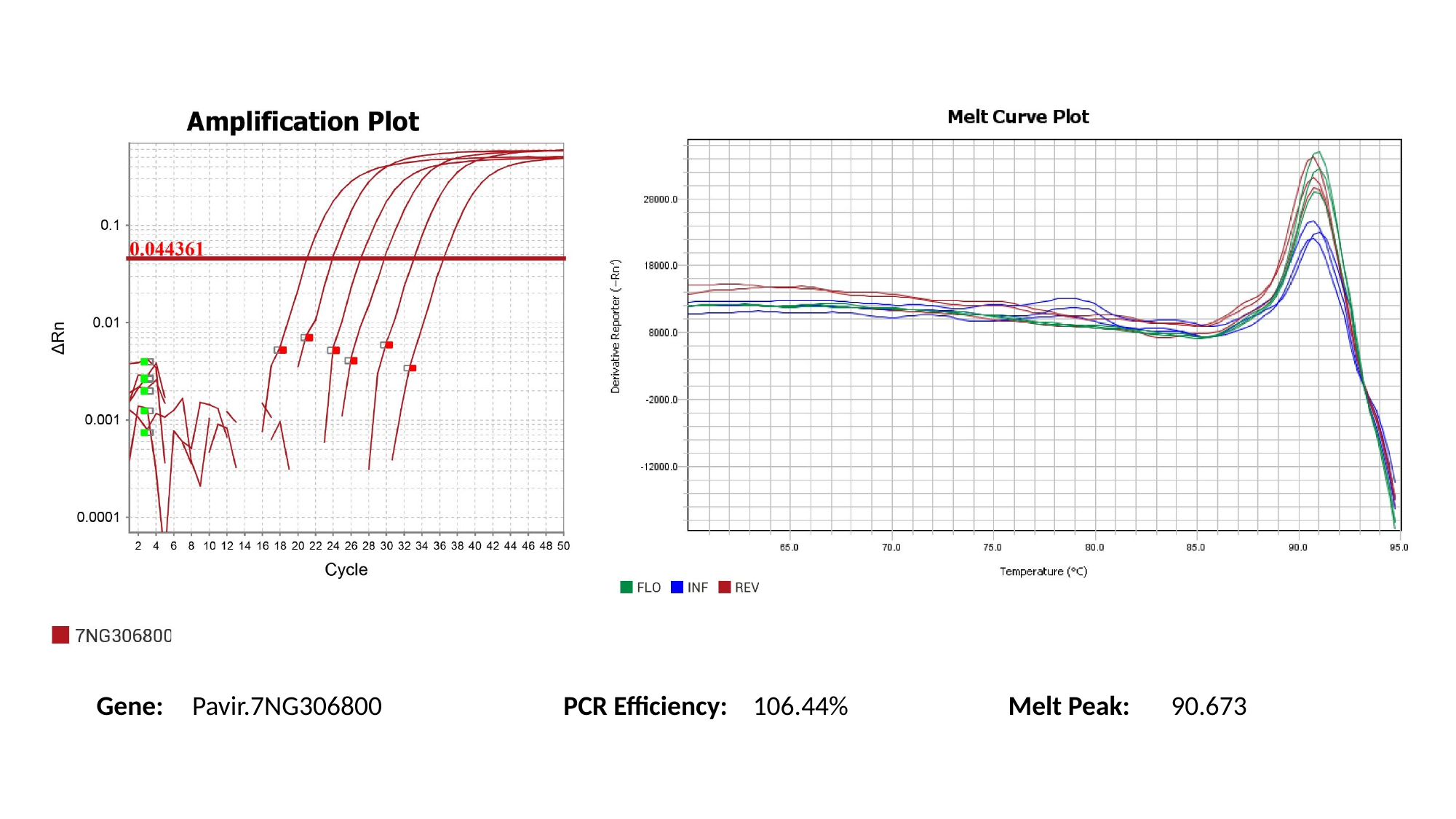

| Gene: | Pavir.7NG306800 | PCR Efficiency: | 106.44% | Melt Peak: | 90.673 |
| --- | --- | --- | --- | --- | --- |

## Slide 11
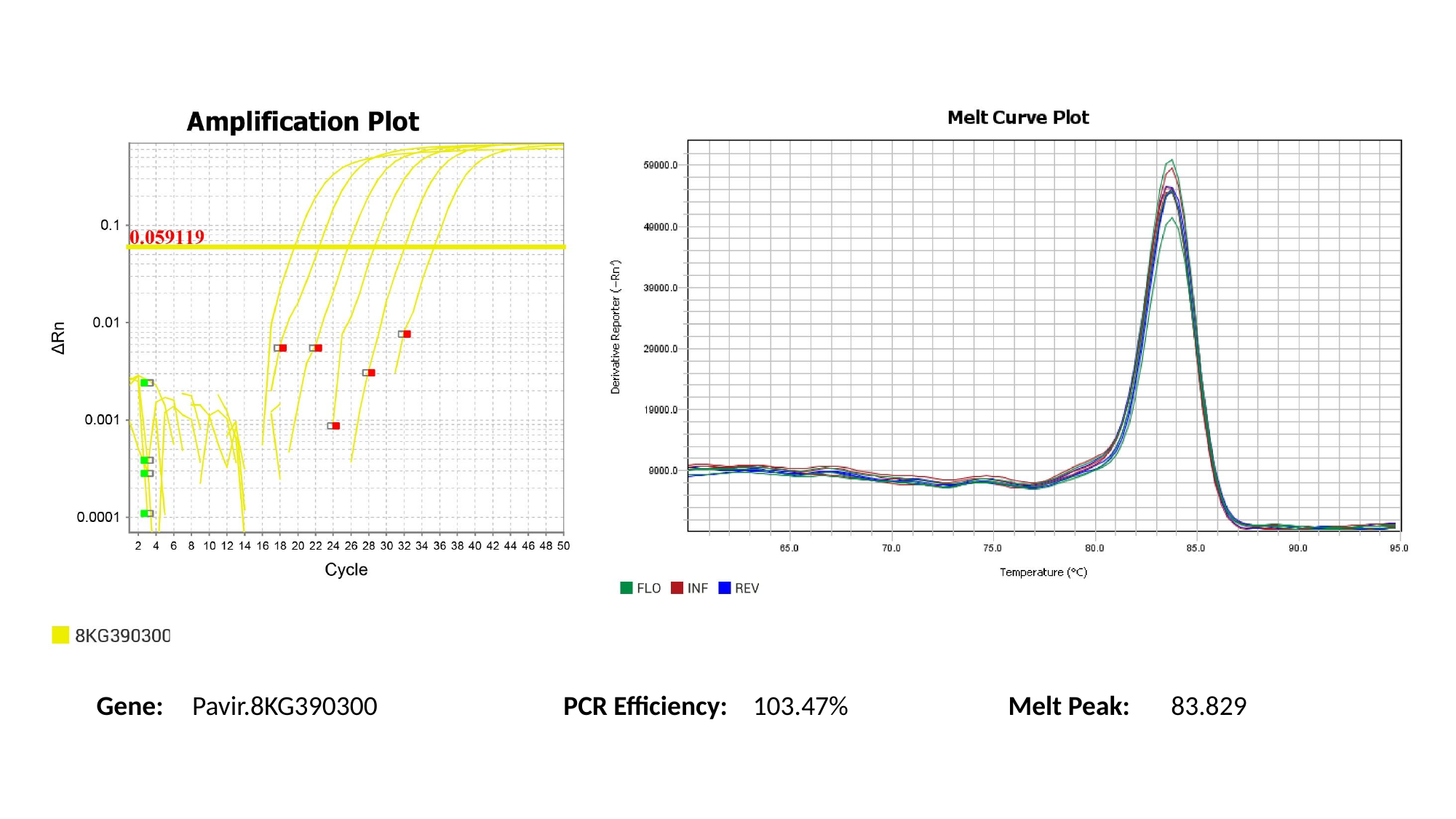

| Gene: | Pavir.8KG390300 | PCR Efficiency: | 103.47% | Melt Peak: | 83.829 |
| --- | --- | --- | --- | --- | --- |

## Slide 12
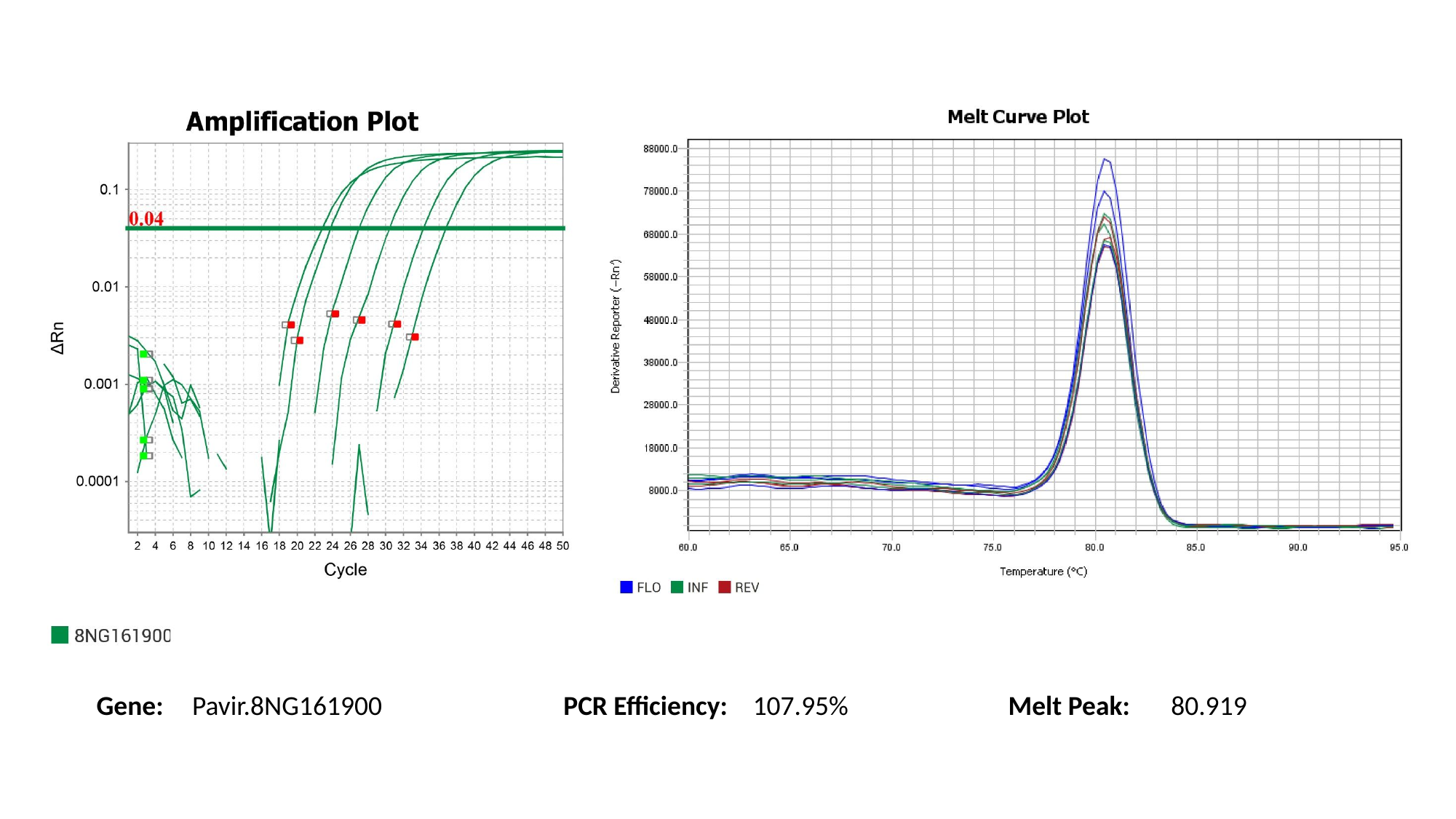

| Gene: | Pavir.8NG161900 | PCR Efficiency: | 107.95% | Melt Peak: | 80.919 |
| --- | --- | --- | --- | --- | --- |

## Slide 13
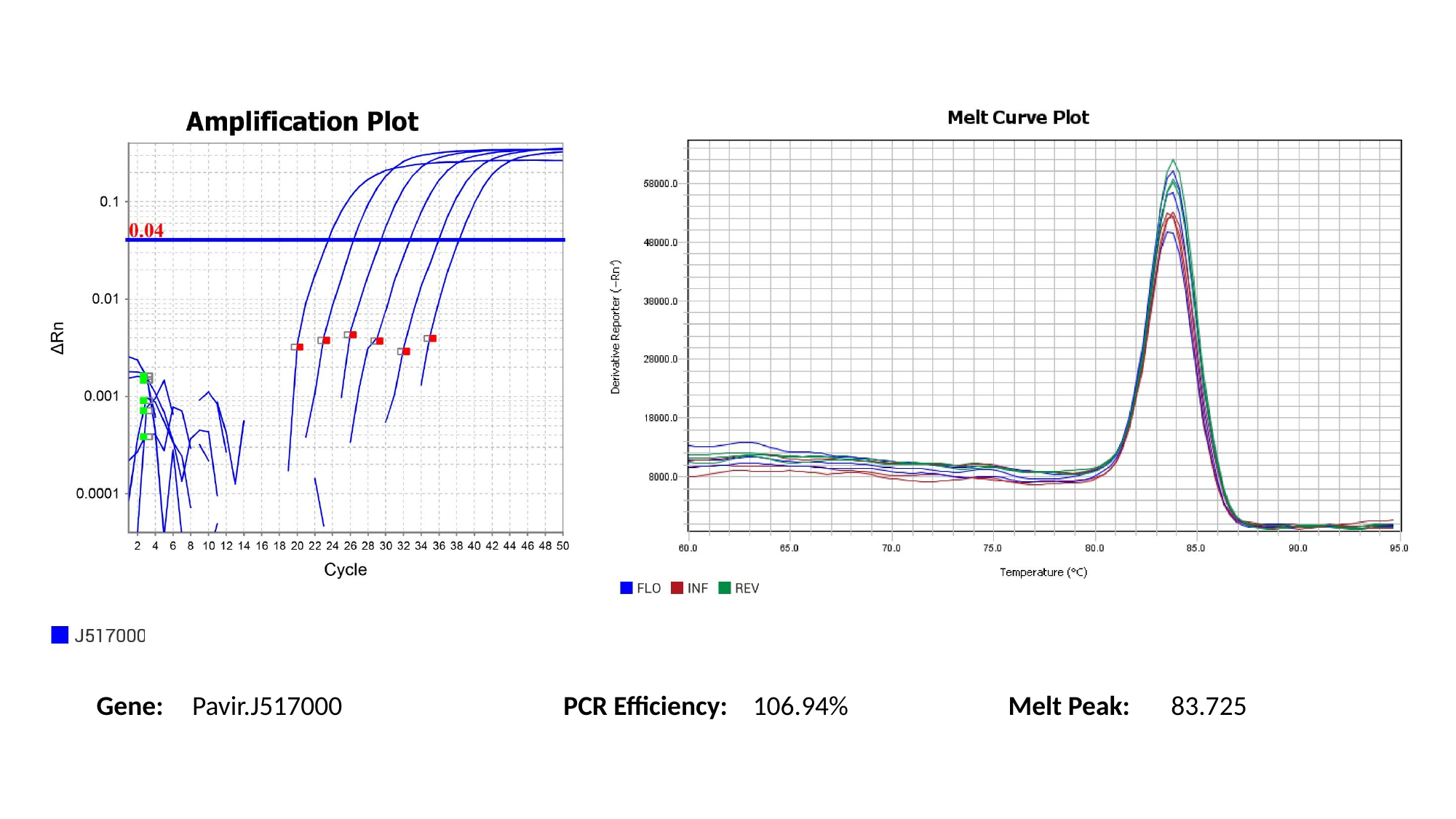

| Gene: | Pavir.J517000 | PCR Efficiency: | 106.94% | Melt Peak: | 83.725 |
| --- | --- | --- | --- | --- | --- |
